# JMY Functions as Actin Nucleation-Promoting Factor and Mediator for p53-Mediated DNA Damage in Porcine Oocytes

**DOI:** 10.1371/journal.pone.0109385

**Published:** 2014-10-03

**Authors:** Zili Lin, Yong-Nan Xu, Suk Namgoong, Nam-Hyung Kim

**Affiliations:** Department of Animal Sciences, Chungbuk National University, Cheongju, Korea; Institute of Zoology, Chinese Academy of Sciences, China

## Abstract

Junction-mediating and regulatory protein(JMY) is a multifunctional protein with roles in the transcriptional co-activation of p53 and the regulation of actin nucleation promoting factors and, hence, cell migration; however, its role in the maturation of porcine oocytes is unclear. In the current study, we investigated functional roles of JMY in porcine oocytes. Porcine oocytes expressed JMY mRNA and protein, and the mRNA expression level decreased during oocyte maturation. Knockdown of JMY by RNA interference decreased the rate of polar body extrusion, validating its role in the asymmetric division of porcine oocytes. JMY knockdown also down-regulated the mRNA and protein levels of actin and Arp2/3. Furthermore, JMY accumulated in the nucleus in response to DNA damage, and JMY knockdown suppressed DNA damage-mediated p53 activation. In conclusion, our results show that JMY has important roles in oocyte maturation as a regulator of actin nucleation-promoting factors and an activator of p53 during DNA damage during DNA damages in porcine oocytes.

## Introduction

In mammalian oocytes, dynamic actin polymerization and reorganization are essential for asymmetric spindle migration, polar body extrusion, and cytokinesis [1], [2]. Actin nucleators, proteins which promote actin polymerization [3], such as formin 2 [4], spire [5], and the Arp2/3 complex [6], [7] play essential roles in oocyte maturation. In addition with actin nucleators, nucleation-promoting factors (NPFs), which bind to and activate the Arp2/3 complex in response to Rho-GTPase signaling [3], are also important for oocyte maturation. For example, Junction-mediating and regulatory protein (JMY), one of actinnucleation-promoting factor [8], [9] is critical for spindle migration and asymmetric division in mouse oocytes [10]. In addition to its role as an actin nucleation promoting factor, JMY was originally identified as a p53 coactivator, localizes to the nucleus during DNA damage [11]–[13]. Upon processing of DNA double-strand breaks (DSBs), JMY forms a complex with strap (stress-responsive activator of p300) and p300, which recruits PRMT5 into a coactivator complex that drives the p53 response [14], [15] It is reported that DNA damage affects JMY activity, which is required for p53 transcriptional activity in MCF-7 cells [15]. Recent studies identify JMY as a target through which Mdm2 regulates p53 activity [12].

Considering dual roles of JMY as DNA damage responsive element and actin nucleation promoting factors in somatic cells, we postulated that JMY could be involved in the DNA damage response in oocytes. The DNA damage response in mammalian oocytes is important to maintain genetic integrity, especially mammalian oocytes stay in prophase in several years before fertilization [16]. In contrast with somatic cells, oocytes progress to the M-phase even after moderate DNA damage, but severe DNA damage can activate the ATM/Chk1-dependent DNA damage checkpoint and cause arrest at prophase [17], indicating the difference of DNA damage response in somatic cell and oocytes.

In the current study, we investigated the roles of JMY as a regulator of actin nucleation-promoting activity and also as an activator of p53 during DNA damage in maturing porcine oocytes.

## Materials and Methods

### In vitro maturation (IVM) of porcine oocytes

Porcine ovaries were collected from gilts at a commercial slaughterhouse and transported to the laboratory in saline maintained at 37°C. Cumulus oocyte complexes (COCs) were aspirated from follicles 2–6 mm in diameter using an 18-gauge needle and syringe. COCs with intact and unexpanded cumulus cells were isolated and cultured in tissue culture medium (TCM)-199 containing 0.1% polyvinyl alcohol (PVA, w/v), 3.05 mM D-glucose, 0.91 mM sodium pyruvate, 0.57 mM cysteine, 10 ng/mL epidermal growth factor (Sigma, St. Louis, MO, USA), 10 IU/mL PMSG (Pregnant Mare Serum Gonadotropin), 10 IU/mL hCG (Human Chorionic Gonadotropin), 75 µg/mL penicillin G, and 50 µg/mL streptomycin sulfate under mineral oil for 44 h at 38.5°C in a humidified atmosphere of 5% CO_2_ (v/v) in air. To induce DNA damage, 25 mg/mL of etoposide (Sigma) was added into the IVM medium.

### Preparation of double-stranded RNA (dsRNA)

To knockdown JMY in porcine oocytes, JMY dsRNA was generated as described previously [18]. Using two primers (Table 1) from bp 2266 to bp 2811 of the porcine JMY gene (XM_003123744.1), a 570-bp fragment containing the T7 promoter was amplified from cDNA from oocytes at the germinal vesicle (GV) stage. The PCR product underwent gel purification, and it was then used as a template for in vitro transcription using the mMessage mMachine T7 transcription kit (Cat. no. AM1344, Ambion, Austin, TX). The T7 promoter at both ends of the PCR product initiated transcription in both directions, resulting in sense and antisense JMY transcripts. After the in vitro transcription reaction, the template DNA was removed by Turbo-DNase (Life Technologies, Foster City, CA, USA), and the product was further purified by phenol–chloroform extraction and isopropanol precipitation. The dsRNA sample was stored at −80°C until use.

**Table 1:** Primers used in this study.

	GenBank Accession	Primer Sequence (5′-3′)	Length (bp)
*JMY* dsRNA	XM_003123744.1	F**:ATTAATACGACTCACTATAGGGAG**AACTGCCTCCCACTGTATCG	570
		R**:ATTAATACGACTCACTATAGGGAG**AGCTCCGTGTTAGAGGGTCT	
*JMY*	XM_003123744.1	F: TTCCGAGACATGCGAGAACT	467
		R: TGCTGCCCATGATGCTTTAC	
*Arp2*	NM_001134354.1	F:CTCACAGAACCTCCTATGAACCC	197
		R:CCCAGCAATATCCAGTCTCCT	
*Arp3*	NM_001134343	F:TAAGGGCAGSACCTGAAGAC	323
		R:CCACTGGGATGACATGAGTG	
*Actin*	AY550069	F:CACGCCATCCTGCGTCTGGA	417
		R:AGCACCGTGTTCGCCTACA	
*P53*	NM_213824	F:TCGTCCTTTGTCCCTTCTCAG	240
		R: CACCACCTCGGTCATGTACTCT	
*GAPDH*	AF017079	F:GGGCATGAACCATGAGAAGT	230
		R: AAGCAGGGATGATGTTCTGG	

*T7 promoter sequences used to generate the JMY dsRNA for knockdown are indicated in bold.

### Microinjection of oocytes with JMY dsRNA

Microinjection of JMY dsRNA into the cytoplasm of oocytes was performed as described previously [18] with the Femtojet constant flow system (Eppendorf AG, Hamburg, Germany) and a Nikon Diaphot ECLIPSE TE300 inverted microscope (Nikon UK Ltd., Kingston upon Thames, Surrey, UK) equipped with a Narishige MM0-202N hydraulic three-dimensional micromanipulator (Narishige Inc., Sea Cliff, NY, USA). Each oocyte was injected with approximately 10 pL (1 µg/uL) of JMY dsRNA, and oocytes were cultured under paraffin oil at 38.5°C. The developmental stage of oocytes was determined by staining with 1 µg/mL of 4′-6-diamidino-2-phenylindole (DAPI) for 10 min. The control oocytes were microinjected with 5–10 picoliter of green fluorescent protein dsRNA. All microinjection experiments were performed at least five independent times, and approximately 100 oocytes were injected in each group.

### Immunofluorescence and confocal microscopy

The denuded porcine oocytes were fixed in 3.7% paraformaldehyde (w/v) in phosphate-buffered saline (PBS) containing 0.1% PVA (w/v) for 30 min at room temperature. Oocytes were washed three times with PBS-0.1% PVA and permeabilized with 1% Triton X-100 (v/v) for 30 min at 37°C, followed by blocking with 1% BSA (w/v) for 1 h. To determine the cellular distribution of proteins, oocytes were incubated overnight at 4°C with an anti-JMY (Cat. no. sc-13020, lot no. E1512, Santa Cruz Biotechnology, Inc., Santa Cruz, CA, USA) antibody diluted 1∶100 in blocking buffer. Alexa Fluor 568 Goat anti-rabbit IgG (Invitrogen, Carlsbad, CA, USA) was used as the secondary antibody. The nuclear status of oocytes was determined by staining with 10 µg/mL of propidium iodide (PI) for 20 min. Following extensive washing, oocytes were mounted between a coverslip and a glass slide. The negative control in each group was incubated only with the secondary antibody and DAPI. Anti-α-tubulin (Sigma), tetramethylrhodamine-labeled phalloidin (Life technology, Foster City, CA, USA), anti-Arp2, anti-γ-H_2_AX antibody, were used for filament actin, Arp2 and γ-H_2_AX staining, respectively. Oocytes were examined under a Zeiss LSM 710 META confocal laser-scanning microscope (Jena, Germany).

### Real-time quantitative PCR

Total RNA was isolated from frozen porcine oocytes with the Dynabeads mRNA DIRECT kit (Dynal Asa, Oslo, Norway) and reverse transcribed into cDNAs with oligo(dT)_12–18_ and SuperScript II reverse transcriptase (Invitrogen, Grand Island, NY, USA). Real-time PCR was performed with the DyNAmo HS SYBR Green qPCR kit (FINNZYMES, Helsinki, Finland) and a CFX96 real-time PCR system (Bio-Rad, Hercules, CA, USA) with the following conditions: 94°C for 30 sec, followed by 40 cycles at 94°C for 30 sec, 60°C for 30 sec, and 72°C for 25 sec. A final extension of 72°C for 5 min was included at the end of the run. The relative gene expression was quantified by normalization to the GAPDH mRNA level using the ΔΔCT method [19]. The PCR primers used for real-time PCR are listed in Table 1.

### Western blot analysis

Western blot analysis was performed as described previously [20]. Briefly, 200 pig oocytes were thawed at room temperature and added to 20 µL of 1×SDS sample buffer [62.5 mM Tris-HCl pH 6.8 at 25°C containing 2% SDS (w/v), 10% glycerol (v/v), 50 mM DTT, and 0.01% bromophenol blue or phenol red (w/v)]. Samples were then heated at 95°C for 5 min. SDS-PAGE was performed using a Criterion precast gel (Bio-Rad) for 2 h at 100 V, followed by electrophoretic transfer onto a polyvinylidene difluoride (PVDF) membrane with the iBlot system (Invitrogen, Grand Island, NY, USA) for 2.5 h at 200 mA and 4°C. Membranes were blocked with 5% nonfat dry milk and incubated overnight with an anti-JMY (sc-13020), anti-p53 (sc-6242, Santa Cruz Biotechnology, Inc. CA, USA) and anti-GAPDH (ab9484, Abcam Inc. Cambridge, MA, USA) antibody diluted in blocking solution containing 0.05% Tween 20 (v/v). Bands were detected by incubation with a secondary antibody conjugated to horseradish peroxidase and chemiluminescence substrate (ECL Plus, GE Healthcare, Pittsburgh, PA, USA).

### Statistical analysis

All percentage data were subjected to arcsine transformation before statistical analysis. The general linear models (GLM) procedure in the SAS program (SAS Institute Inc., NC, USA) was used to analyze the data from all experiments. Differences with a *p*<0.05 were considered significant. For fluorescence intensity statistics, 10×10 pixels in different areas of 10 oocytes were analyzed by ZEN 2009 software.

## Results

### Expression and subcellular localization of JMY in porcine oocytes

**Figure 1:**
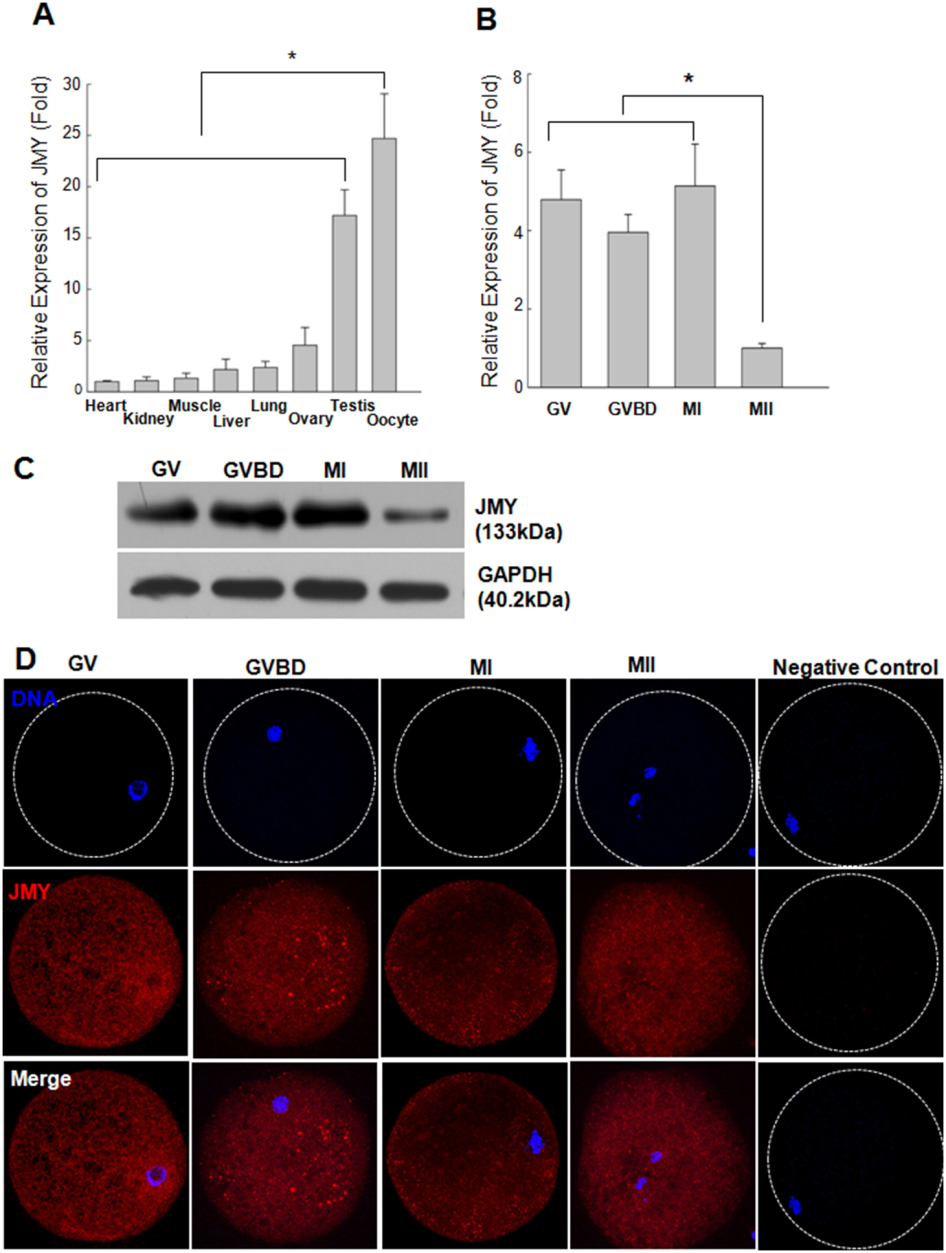
Expression of JMY in different tissues and during porcine oocytes meiotic maturation. A, B: The relative expression of JMY in different tissues (A) and oocytes (B) were measured by quantitative real-time PCR. mRNA levels were normalized to those in heart. Values represent mean ± s.e.m. *p<0.05, n = 3. C: Protein levels of JMY in maturing oocytes measured by western blotting. Two hundreds oocytes were used per lane. D: Subcellular localization of JMY during porcine oocytes meiotic maturation as revealed by immunofluorescence staining. Red: JMY, Blue: chromatin.

Although JMY functions in oocyte maturation and early embryonic development in the mouse [10], [21], [22], its role in other species, including the pig, is unclear. Thus, we examined JMY expression in several porcine tissues and its subcellular localization in the porcine oocytes. As shown in Figure 1A, JMY expression was detected in all examined tissues, with highest expression in the testis and oocytes (*p*<0.05). During oocyte maturation, JMY mRNA (Fig. 1B) and protein (Fig. 1C) were detected at GV, germinal vesicle breakdown (GVBD), and metaphase I (MI) stages; however, JMY expression was lowest at the MII stage. By immunostaining, JMY was found predominantly in the cytoplasm as fragmented and punctuated foci surrounding the germinal vesicle of oocytes at the GV stage (Fig. 1C). After the GVBD stage, its intensity decreased, and JMY was found near the cortex (i.e., the spindle) of oocytes. These results indicate that porcine oocytes express JMY, and that its subcellular localization changes during oocyte maturation.

### Knockdown of JMY disrupts oocytes meiotic maturation

**Figure 2:**
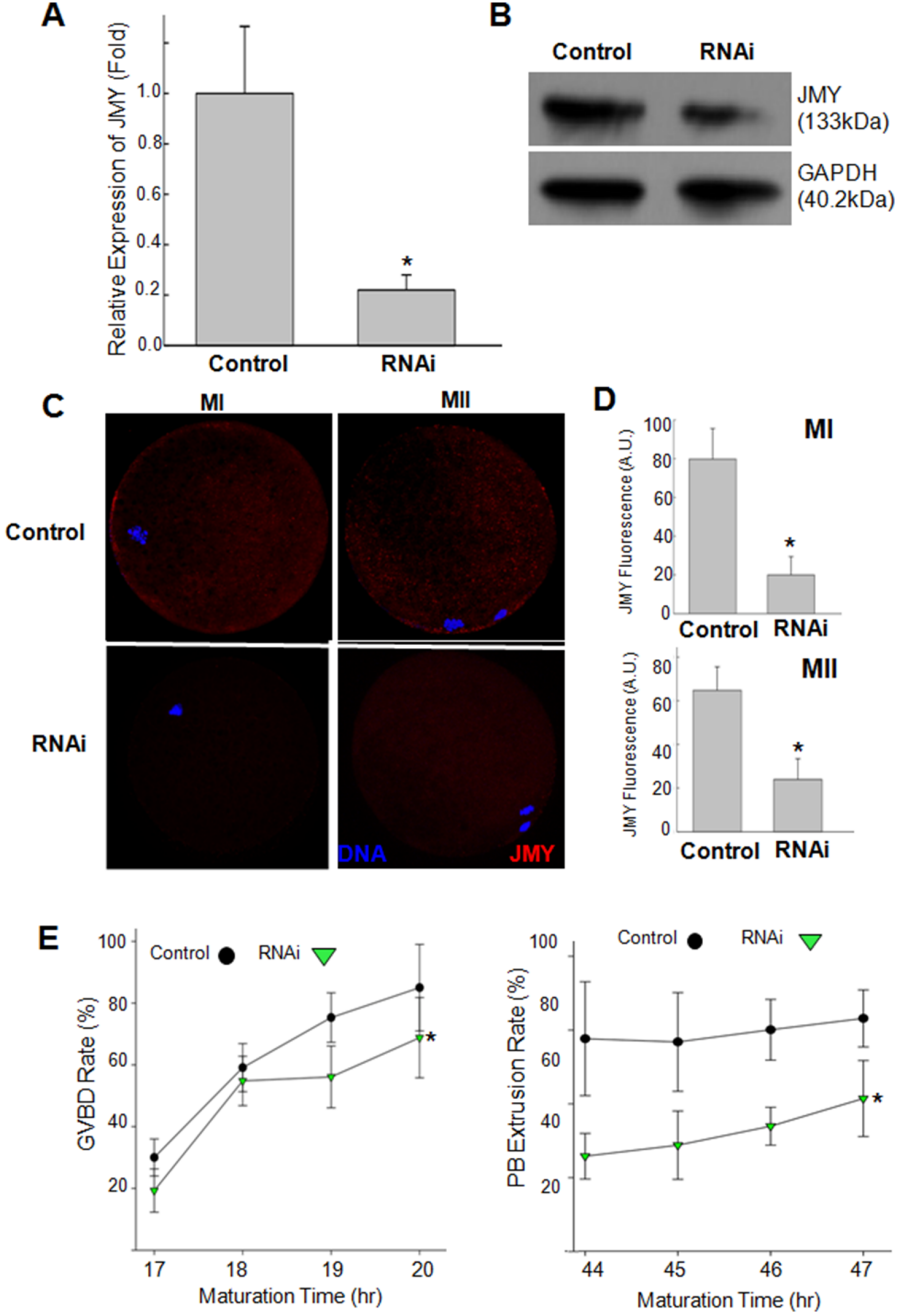
Knockdown of JMY during meiotic maturation. **A–D**: GV oocytes injected with dsRNAs or control were cultured for 44 hours. Knockdown of JMY mRNA was determined by RT-PCR (**A**) and Western blotting (**B**). Subcellular localization (**C**) and quantitized fluorescence intensity (**D**) of JMY fluorescence of dsRNA or control injected oocytes measured at MI (20 h after culture) and MII (44 h after culture) stages respectively. **E**: Germinal vesicle breakdown (GVBD) and polar body extrusion (PBE) rates of JMY dsRNA induced oocytes. Red: JMY, Blue: chromatin. Values represent mean ± s.e.m. *p<0.05, n = 5.

To determine the role of JMY in porcine oocyte maturation, we performed knockdown experiments by injecting porcine JMY dsRNA. After knockdown, the JMY mRNA level in oocytes decreased to 23.0±5.9% of that of the control (Fig. 2A). By Western blotting (Fig. 2B) and immunofluorescence staining (Fig. 2C), we confirmed that JMY protein level also decreased by dsRNA injection. Thereafter, the effects of JMY knockdown on the rates of GVBD and polar body extrusion in oocytes were examined. The rates of GVBD and polar body extrusion rate decreased after JMY knockdown compared with the control (Fig. 2E and 2F).

### Knockdown of JMY disturbs the level of tubulin, actin and Arp2/3

**Figure 3:**
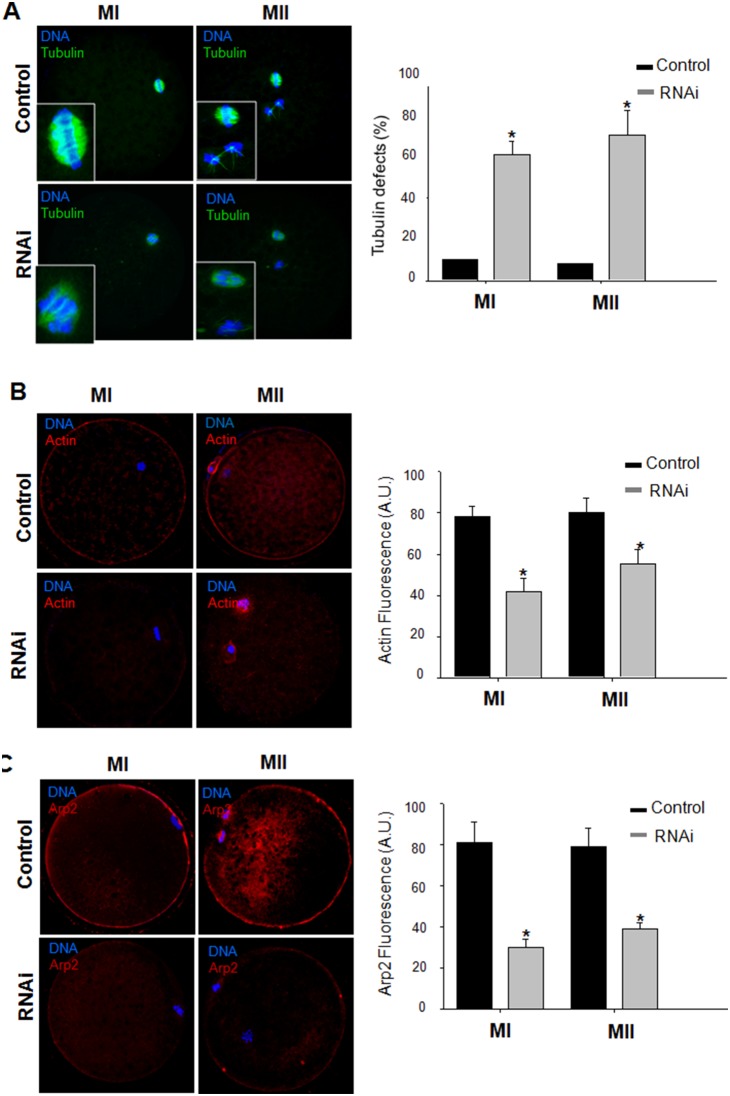
Actin, α-tubulin and Arp2 expression after knockdown of JMY in porcine oocytes. A: Representative images of spindle defects in JMY knockdown oocytes at MI (20 h after culture) and MII (44 h after culture) stages are shown. Spindle was stained with anti-α-tubulin antibody (Green) and DNA was stained using Hoechest 33342 (Blue). B: Abnormal distribution of actin in control and dsRNA microinjection groups of oocytes at MI (20 h after culture) and MII (44 h after culture) stages. Actin(Red), DNA(Blue). Actin fluorescences were measured and quantitized (n = 6). Values represent mean ± s.e.m, *, *p*<0.05. C: Arp2 localization (left panel) and fluorescence intensity (right panel) in porcine oocytes at at MI (20 h after culture) and MII (44 h after culture) stages. Values represent mean ± s.e.m, *, *p*<0.05.

The effect of JMY knockdown on the localization of α-tubulin, actin and the Arp2/3 complex was investigated. We found that the knockdown of JMY severely disturbed the spindle formation (Fig. 3A) As shown in Figure 3B, there was a decrease in the cortical actin level in JMY-silenced oocytes at MI and MII stages compared with the control. This defect was observed in >20% of oocytes injected with JMY dsRNA. In agreement with previously reported results in mouse oocytes [21], JMY knockdown disrupted the localization of Arp2 at the cortex and decreased its fluorescence intensity in cytoplasm by 40% compared with the control at MI and MII stage (Fig. 3C).

### DNA damage results in the nuclear localization of JMY

**Figure 4:**
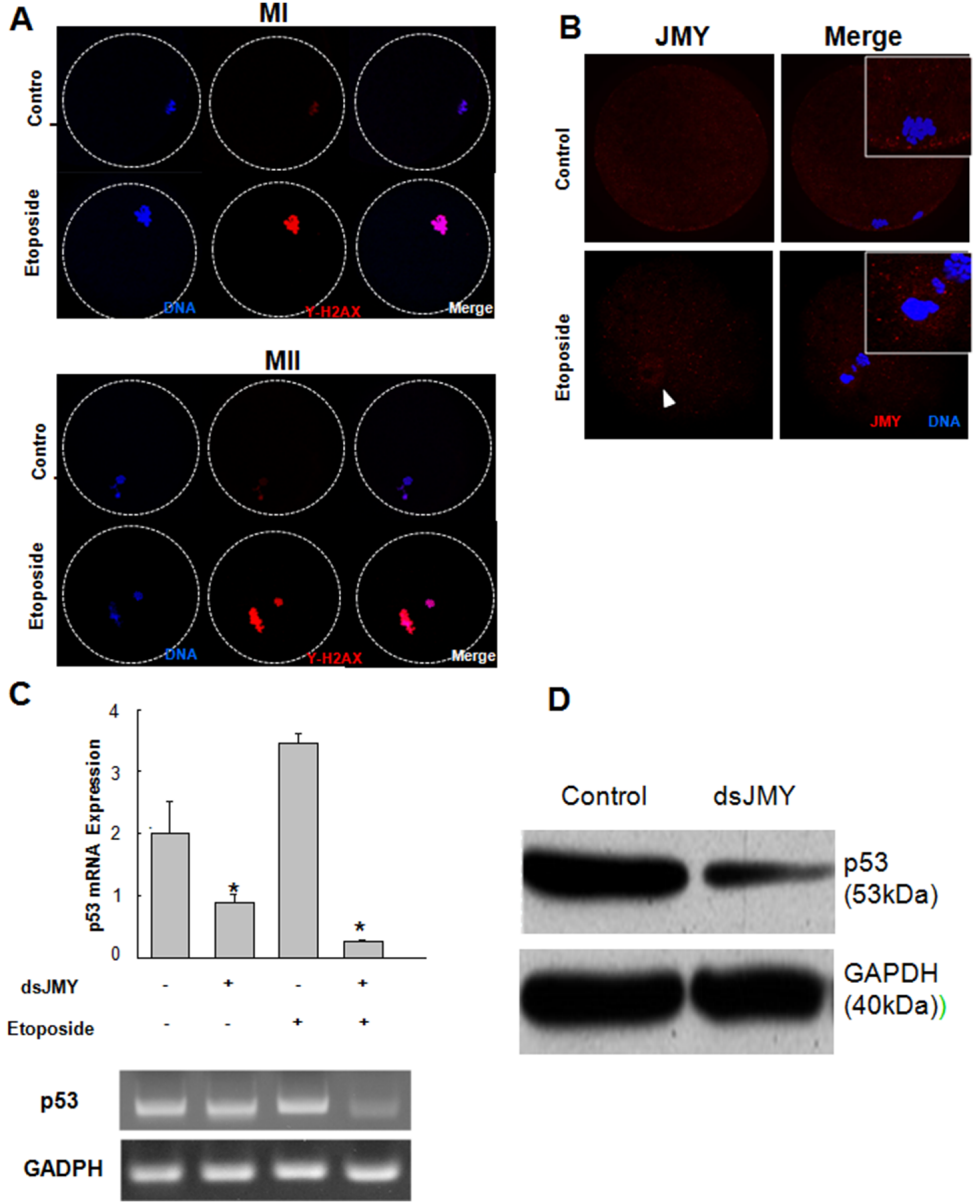
JMY involved in DNA damage responses in maturing porcine oocyte. **A**: Typical staining of γ-H2AX in porcine oocytes before and after the treatment with etoposide (25 mg/mL) at MI (A) and MII (B) stage. Red, γ-H2AX; blue, chromatin. **B**: JMY expression in porcine oocytes on 44 h after etoposide. Note that JMY is localized at nucleus in etoposide treated group (indicated in the arrow). Red, JMY; blue, chromatin. Values represent mean ± s.e.m, *p<0.05, n = 3. **C–D**: p53 expression affected by JMY knockdown and/or etoposide treatment. Porcine oocytes were treated with JMY dsRNA and/or etoposide (25 mg/mL) and expression levels of p53 were quantified by RT-PCR (**C**) or western blotting (**D**). GADPH expression was used as internal control.

DSBs induced by etoposide, a topoisomerase 2 inhibitor [23], [24], can be quantified indirectly by the increase in the phosphorylated histone H2AX (γ-H2AX) level [25]. In light of the role of JMY in DNA damage and oxidative stress [11], [12], [26], [27], and JMY transported into the nucleus, we investigated the relationship between γ-H2AX localization and JMY function in etoposide-treated oocytes. After treatment of oocytes with etoposide for 28 and 44 h, there was a decrease in the γ-H2AX immunostaining signal compared with the control (Fig. 4A), indicating that etoposide induced DSBs in oocytes. Furthermore, JMY was found predominantly at the MII in etoposide-treated oocytes (Fig. 4B), while it was present in the cytoplasm in control oocytes. These results illustrate that DNA damage induces JMY translocation into the nucleus in porcine oocytes.

Lastly, we examined the ability of JMY to activate p53 transcription in oocytes. As shown in Figure 4C, microinjection of JMY dsRNA decreased p53 expression. We also investigated the level of p53 in etoposide-treated oocytes because the formation of DSBs by etoposide can upregulate p53 expression in somatic cells [23]. When porcine oocytes were microinjected with JMY dsRNA and then treated with etoposide, p53 expression was suppressed. These results support the contention that JMY is a p53 transcriptional coactivator in oocytes.

## Discussion

Originally identified as a coactivator of p53 [11], JMY has roles in DNA damage [11], [12], cell motility [3], and oocyte maturation [13], [20]. Recently JMY was identified as actin nucleating promoting factor [8]. These multifunctional roles of JMY hindered understanding the exact functional roles in oocyte maturation. To understand exact functional roles of JMY in oocyte maturation, we investigated its functions in the regulation of actin NPFs and the activation of p53 during DNA damage.

The expression and subcellular localization of JMY in the porcine was comparable with that of the mouse, and its knockdown decreased the rate of polar body extrusion in oocytes. These abnormalities may have been caused by defects in actin nucleation function. Furthermore, JMY knockdown decreased the protein levels of actin and Arp2/3, indicating that JMY regulates actin and Arp2/3 expression. However, the precise molecular mechanisms for these phenomena are unclear. One possible explanation is that JMY directly affects actin and Arp2/3 expression as a transcriptional coactivator. The binding of actin to JMY can affect the cellular localization of JMY [9]; thus, cellular levels of actin can control the translocation of JMY into the nucleus and translocated JMY may affect expression level of actin and Arp2/3. But detailed involvement and mechanism of JMY in actin and Arp2/3 expression remained to be investigated.

**Figure 5:**
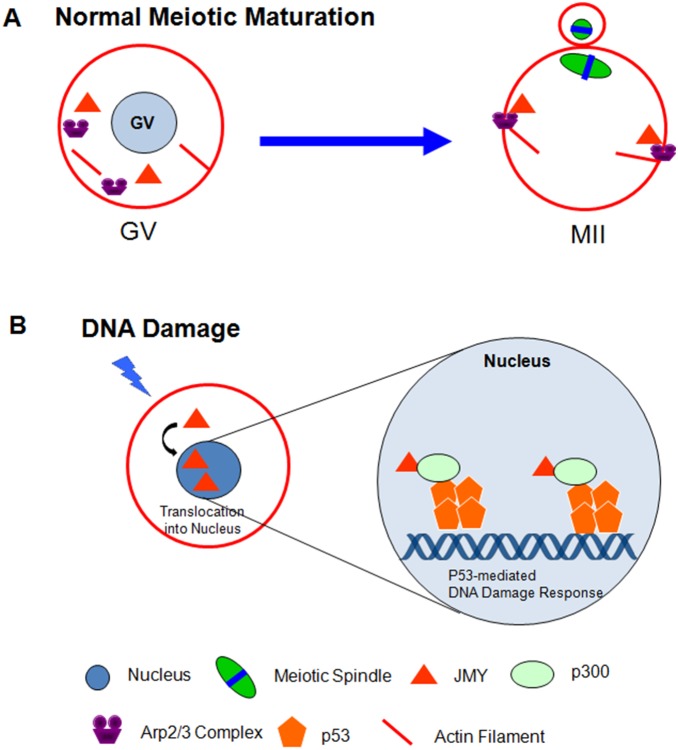
A working model for roles of JMY in porcine oocytes maturation and DSB pathway. A: JMY is involved in oocyte maturation and asymmetric spindle migration, presumably via its actin nucleation promoting activity. B: Upon DNA damage, JMY have a tendency to nuclear accumulation, which is recruited to the promoters of p53 target genes and facilitates the p53 response.

In light of the canonical role of JMY as a p53 cofactor, we investigated the relationship between JMY function and p53 activation in response to DNA damage in porcine oocytes. We employed etoposide, a DNA topoisomerase 2 inhibitor, to introduce DSBs [24]. After treatment of oocytes with etoposide, there was a drastic increase in the JMY immunostaining signal in the nucleus, revealing that JMY shuttles between the cytoplasm and nucleus in oocytes, similar to somatic cells [9]. JMY knockdown also decreased the p53 mRNA level. Moreover, increased expression of p53 upon etoposide treatment effectively suppressed by JMY knockdown, suggesting a role for JMY in p53-mediated DNA damage in oocytes in addition with those of nucleation promoting factor, as illustrated in the Figure 5.

Mammalian oocytes are particularly vulnerable to DNA damage, especially in human, because they must remain in a dormant stage for more than 40 years until maturation [16]. Thus, the detection and repair of DNA damage are critical for the maintenance of oocytes integrity and genetic information. In somatic cells, DSBs normally cause G2 arrest [28]. In oocytes, DNA damage-mediated prophase arrest have been reported in high concentration of DNA damaging agent including etoposide, doxorubicin or neocarzinostatin [17], [29], although DNA damage detection evaluated by the presence of γ-H2AX are very sensitive as similar with somatic cell [17], indicating the presence of DSB responsive machinery in prophase-arrested oocytes.

Similar to the p53-dependent role of JMY in NIH 3T3 Ras or MCF-7 cells [12], JMY may act as a ‘damage sensor’ to relay signals to the nucleus. Recently, JMY translocation into the nucleus has been shown to involve binding of monomeric actin [9], indicating that cytoplasmic unpolymerized actin may regulate JMY translocation. Furthermore, F-actin assembly in cells is regulated by protein MICAL (Molecule Interacting with CasL), which oxidizes actin with hydrogen peroxide, prevents actin polymerization, and increases the unpolymerized actin level in the presence of oxidative stress [30]. JMY may link oxidative stress with DNA damage responses in oocytes, but the exact mechanism is open to be investigated.

In conclusion, our results show that JMY has important roles in oocyte maturation as a regulator of actin NPFs and an activator of p53 during DNA damage.

## References

[1] Sun QY, Schatten H (2006) Regulation of dynamic events by microfilaments during oocyte maturation and fertilization. Reproduction 131: 193–205. 16452714 10.1530/rep.1.00847

[2] Yi K, Li R (2012) Actin cytoskeleton in cell polarity and asymmetric division during mouse oocyte maturation. Cytoskeleton (Hoboken) 69: 727–737. 22753278 10.1002/cm.21048

[3] Pollard TD, Cooper JA (2009) Actin, a central player in cell shape and movement. Science 326: 1208–1212. 19965462 10.1126/science.1175862 PMC3677050

[4] Leader B, Lim H, Carabatsos MJ, Harrington A, Ecsedy J, et al (2002) Formin-2, polyploidy, hypofertility and positioning of the meiotic spindle in mouse oocytes. Nat Cell Biol 4: 921–928. 12447394 10.1038/ncb880

[5] Pfender S, Kuznetsov V, Pleiser S, Kerkhoff E, Schuh M (2011) Spire-type actin nucleators cooperate with Formin-2 to drive asymmetric oocyte division. Curr Biol 21: 955–960. 21620703 10.1016/j.cub.2011.04.029 PMC3128265

[6] Sun SC, Wang ZB, Xu YN, Lee SE, Cui XS, et al (2011) Arp2/3 complex regulates asymmetric division and cytokinesis in mouse oocytes. PLoS One 6: e18392. 21494665 10.1371/journal.pone.0018392 PMC3072972

[7] Yi K, Unruh JR, Deng M, Slaughter BD, Rubinstein B, et al (2011) Dynamic maintenance of asymmetric meiotic spindle position through Arp2/3-complex-driven cytoplasmic streaming in mouse oocytes. Nat Cell Biol 13: 1252–1258. 21874009 10.1038/ncb2320 PMC3523671

[8] Zuchero JB, Coutts AS, Quinlan ME, Thangue NB, Mullins RD (2009) p53-cofactor JMY is a multifunctional actin nucleation factor. Nat Cell Biol 11: 451–459. 19287377 10.1038/ncb1852 PMC2763628

[9] Zuchero JB, Belin B, Mullins RD (2012) Actin binding to WH2 domains regulates nuclear import of the multifunctional actin regulator JMY. Mol Biol Cell 23: 853–863. 22262458 10.1091/mbc.E11-12-0992 PMC3290644

[10] Sun SC, Sun QY, Kim NH (2011) JMY is required for asymmetric division and cytokinesis in mouse oocytes. Mol Hum Reprod 17: 296–304. 21266449 10.1093/molehr/gar006

[11] Shikama N, Lee CW, France S, Delavaine L, Lyon J, et al (1999) A novel cofactor for p300 that regulates the p53 response. Mol Cell 4: 365–376. 10518217 10.1016/s1097-2765(00)80338-x

[12] Coutts AS, Boulahbel H, Graham A, La Thangue NB (2007) Mdm2 targets the p53 transcription cofactor JMY for degradation. EMBO Rep 8: 84–90. 17170761 10.1038/sj.embor.7400855 PMC1796743

[13] Coutts AS, Pires IM, Weston L, Buffa FM, Milani M, et al (2011) Hypoxia-driven cell motility reflects the interplay between JMY and HIF-1alpha. Oncogene 30: 4835–4842. 21625218 10.1038/onc.2011.188

[14] Demonacos C, Krstic-Demonacos M, La Thangue NB (2001) A TPR motif cofactor contributes to p300 activity in the p53 response. Mol Cell 8: 71–84. 11511361 10.1016/s1097-2765(01)00277-5

[15] Demonacos C, Krstic-Demonacos M, Smith L, Xu D, O'Connor DP, et al (2004) A new effector pathway links ATM kinase with the DNA damage response. Nat Cell Biol 6: 968–976. 15448695 10.1038/ncb1170

[16] Carroll J, Marangos P (2013) The DNA damage response in mammalian oocytes. Front Genet 4: 117. 23805152 10.3389/fgene.2013.00117 PMC3690358

[17] Marangos P, Carroll J (2012) Oocytes progress beyond prophase in the presence of DNA damage. Curr Biol 22: 989–994. 22578416 10.1016/j.cub.2012.03.063

[18] Li YH, Kang H, Xu YN, Heo YT, Cui XS, et al (2013) Greatwall kinase is required for meiotic maturation in porcine oocytes. Biol Reprod 89: 53. 23843240 10.1095/biolreprod.113.109850

[19] Livak KJ, Schmittgen TD (2001) Analysis of relative gene expression data using real-time quantitative PCR and the 2(-Delta Delta C(T)) Method. Methods 25: 402–408. 11846609 10.1006/meth.2001.1262

[20] Lin ZL, Li YH, Xu YN, Wang QL, Namgoong S, et al (2014) Effects of growth differentiation factor 9 and bone morphogenetic protein 15 on the in vitro maturation of porcine oocytes. Reprod Domest Anim 49: 219–227. 24313324 10.1111/rda.12254

[21] Liu J, Wang QC, Wang F, Duan X, Dai XX, et al (2012) Nucleation promoting factors regulate the expression and localization of Arp2/3 complex during meiosis of mouse oocytes. PLoS One 7: e52277. 23272233 10.1371/journal.pone.0052277 PMC3525642

[22] Wang QC, Liu J, Wang F, Duan X, Dai XX, et al (2013) Role of nucleation-promoting factors in mouse early embryo development. Microsc Microanal 19: 559–564. 23552571 10.1017/S1431927613000032

[23] Fritsche M, Haessler C, Brandner G (1993) Induction of nuclear accumulation of the tumor-suppressor protein p53 by DNA-damaging agents. Oncogene 8: 307–318. 8426740

[24] Baldwin EL, Osheroff N (2005) Etoposide, topoisomerase II and cancer. Curr Med Chem Anticancer Agents 5: 363–372. 16101488 10.2174/1568011054222364

[25] Tanaka T, Halicka HD, Traganos F, Seiter K, Darzynkiewicz Z (2007) Induction of ATM activation, histone H2AX phosphorylation and apoptosis by etoposide: relation to cell cycle phase. Cell Cycle 6: 371–376. 17297310 10.4161/cc.6.3.3835

[26] Wrighton KH (2009) JMY: actin up in cell motility. Nat Rev Mol Cell Biol 10: 304. 10.1038/nrm2678 19391193

[27] Roadcap DW, Bear JE (2009) Double JMY: making actin fast. Nat Cell Biol 11: 375–376. 19337319 10.1038/ncb0409-375 PMC2745592

[28] Harrison JC, Haber JE (2006) Surviving the breakup: the DNA damage checkpoint. Annu Rev Genet 40: 209–235. 16805667 10.1146/annurev.genet.40.051206.105231

[29] Yuen WS, Merriman JA, O'Bryan MK, Jones KT (2012) DNA double strand breaks but not interstrand crosslinks prevent progress through meiosis in fully grown mouse oocytes. PLoS One 7: e43875. 22928046 10.1371/journal.pone.0043875 PMC3425511

[30] Hung RJ, Pak CW, Terman JR (2011) Direct redox regulation of F-actin assembly and disassembly by Mical. Science 334: 1710–1713. 22116028 10.1126/science.1211956 PMC3612955

